# Modification of the Polymer of a Bone Cement with Biodegradable Microspheres of PLGA and Loading with Daptomycin and Vancomycin Improve the Response to Bone Tissue Infection

**DOI:** 10.3390/polym14050888

**Published:** 2022-02-23

**Authors:** Joaquin García-García, Galo Azuara, Oscar Fraile-Martinez, Cielo García-Montero, Miguel Angel Álvarez-Mon, Sara Ruíz-Díez, Melchor Álvarez-Mon, Julia Buján, Natalio García-Honduvilla, Miguel A. Ortega, Basilio De la Torre

**Affiliations:** 1 Service of Orthopedic Surgery of University Hospital Principe de Asturias, 28805 Alcalá de Henares, Madrid, Spain; joaquingarciagarcia@gmail.com; 2 Service of Traumatology of University Hospital of Guadalajara, 19002 Guadalajara, Spain; galoazu@hotmail.com; 3 Departments of Medicine and Medical Specialities, Faculty of Medicine and Health Sciences, University of Alcalá, Alcalá de Henares, 28801 Madrid, Spain; oscarfra.7@hotmail.com (O.F.-M.); cielo.gmontero@gmail.com (C.G.-M.); maalvarezdemon@icloud.com (M.A.Á.-M.); sruizdiez16@gmail.com (S.R.-D.); mademons@gmail.com (M.Á.-M.); mjulia.bujan@uah.es (J.B.); natalio.garcia@uah.es (N.G.-H.); 4 Ramón y Cajal Institute of Sanitary Research (IRYCIS), 28034 Madrid, Spain; 5 Immune System Diseases-Rheumatology, Oncology Service an Internal Medicine, University Hospital Príncipe de Asturias, (CIBEREHD), 28806 Alcala de Henares, Spain; 6 Department of Surgery, Medical and Social Sciences, Faculty of Medicine and Health Sciences, University of Alcalá, Alcalá de Henares, 28801 Madrid, Spain; bjtorre@gmail.com; 7 Service of Traumatology of University Hospital Ramón y Cajal, 28034 Madrid, Spain

**Keywords:** preclinical model, bone, polymer-based construct of poly (lactic-*co*-glycolic acid) (PLGA), infection

## Abstract

Chronic infections are one of the most serious adverse outcomes of prosthetic surgery. Prosthetic revision surgery using a bone cement loaded with antibiotics between the two stages of the surgery is commonly performed. However, this method often fails to reach the minimum inhibitory concentration and promotes antibiotic resistance, thus emphasizing the need for improving the current available therapies. Materials and methods: In this study, we performed a study of the in vivo response of a polymer-based construct of poly (lactic-*co*-glycolic acid) (PLGA) in the solid phase of Palacos R^®^ in combination with vancomycin, daptomycin, and/or linezolid. To test its effectiveness, we applied an in vivo model, using both histological and immunohistochemical analyses to study the bone tissue. Results: The presence of PLGA in the combination of vancomycin with daptomycin showed the most promising results regarding the preservation of bone cytoarchitecture and *S. aureus* elimination. Conversely, the combination of vancomycin plus linezolid was associated with a loss of bone cytoarchitecture, probably related to an increased macrophage response and inefficient antimicrobial activity. Conclusions: The modification of Palacos R^®^ bone cement with PLGA microspheres and its doping with the antibiotic daptomycin in combination with vancomycin improve the tissue response to bone infection.

## 1. Introduction

Chronic infection is one of the most serious complications of prosthetic surgery, with a prevalence of 0.3–2.2% in primary surgery [1] and 3–4% in revision surgery [2]. Prosthetic infection is a therapeutic challenge due to the appearance of biofilms, made by microorganisms that are enveloped and protected in them, which strongly adhere to the surface of the implant [3]. Several strategies can be applied to treat prosthetic infections, mainly depending on the time of onset of the infection. For chronic infections, prosthetic revision surgery performed in two stages has been the most commonly used method, using a bone cement spacer between the first and second stages [4,5]. This bone cement spacer is loaded with antibiotics, whose release results in much higher local concentrations than can be achieved through intravenous injection. This release is higher in the first few days but persists up to several weeks, in many cases at concentrations below the minimum inhibitory concentration (MIC) of the microbe, which has been associated with the development of antibiotic resistance, the colonization of spacers, and, therefore, therapeutic failure [6,7].

Approximately 20% of *Staphylococcus aureus* and 80% of *Staphylococcus epidermidis* isolated from prosthetic infections are resistant to methicillin [8]. The antibiotic of choice for the treatment of methicillin-resistant staphylococcal infections is vancomycin [9]. The growing abundance of multidrug-resistant bacteria makes it necessary to incorporate new antibiotics into bone cement. Daptomycin and linezolid are the two main alternatives to vancomycin in cases of bacterial resistance [10], having demonstrated good release kinetics and antibacterial properties in in vitro cement [11]. Daptomycin is a calcium-dependent cyclic lipopeptide. It was the first in class of a new group of calcium-dependent membrane-binding lipopeptides [12]. Linezolid is the first member of the class of oxazolidinones. The compound is a synthetic antibiotic that inhibits bacterial protein synthesis by binding to rRNA [13].

Prosthetic infection affects the surrounding bone tissue and its remodeling capacity, which are difficult to evaluate in clinical practice. Experimental animal models are necessary to investigate these issues. There are widely used histological scales created by different authors [14,15], but they evaluate very specific aspects of the bone histoarchitecture and exhibit great variability depending on the strain of the bacteria, the inoculum, and the selected animal model. For this reason, we developed a new histological staging system that can assess bone tissue in a complete way, providing a global picture of the conservation and remodeling of bone histoarchitecture in the presence of infection and under the influence of bone cement [16,17].

Our study group managed to increase the release of vancomycin, daptomycin, and linezolid in vitro, significantly improving the kinetics of their temporary release by incorporating biodegradable microparticles of poly(lactic-*co*-glycolic acid) (PLGA) in the solid phase of Palacos R cement ^®^ [11]. Using these developed cements and applying the new histological staging system that we developed [16], in this paper, we conducted the present study to (1) assess the histological behavior of the modified cement loaded with vancomycin, daptomycin, and/or linezolid in a rabbit model of osteomyelitis; (2) assess the tissue distribution of bacteria; and (3) analyze the presence of macrophages around the different formulations.

## 2. Materials and Methods

### 2.1. Formulations of Bone Cement

The formulations of acrylic bone cements were loaded with two new-generation antibiotics, linezolid (Pfizer, Peapack, NJ, USA) and daptomycin (Cubist Pharmaceutical Incorporation, Kenilworth, NJ, USA), individually or in combination with vancomycin. The composition of the solid phase of commercial bone cement Palacos R^®^ (Heraeus Medical, Wehrheim, Germany) was modified by incorporating microspheres of a biodegradable copolymer, PLGA, as described in our previous study [11].

### 2.2. Titanium Rods Coated with Hydroxyapatite Contaminated with S. aureus

The rods used in the experimental model were made of grade 4 titanium and measured 3 mm in diameter and 10 mm in length. They had a coating with a thickness less than 150 microns, made with a solution of poly(methyl methacrylate) (PMMA) in acetone (10%) and crude hydroxyapatite (HA) in suspension at a ratio of PMMA:HA of 1:1.2 [16].

A clinical strain of *Staphylococcus aureus* was selected, which was sensitive to vancomycin (MIC: 1 μg/mL), linezolid (MIC: 2 μg/mL), daptomycin (MIC: 0.5 μg/mL), gentamicin (MIC: 0.5 μg/mL), rifampicin (MIC: 0.5 μg/mL), and clindamycin (MIC: 0.25 μg/mL) and resistant to other antibiotics, including cloxacillin, cephalosporins, and quinolones. A suspension was prepared with an inoculum of the germ equivalent to 1.2 × 10^9^ colony-forming units/mL. In each tube with this suspension, a titanium rod coated with PMMA:HA was introduced for 24 h to achieve contamination. The presence of many cocci adhering to the rod after extraction from the suspension was observed by scanning electron microscopy [16]. This rod became the implant contaminated with methicillin-resistant *S. aureus* (MRSA) that we introduced into the bone tissue of the rabbit [16,17].

### 2.3. Animal Experimental Model

As the experimental animal, we used rabbits *(Oryctolagus cuniculus)* of the New Zealand variety, male, with a weight between 2.5 and 3.2 kg and an age of 12 weeks at the beginning of the study. They had physeal cartilage in the distal femur, as they were not skeletally mature [17]. The animals were divided into six study groups (1–6) according to the use of the commercial cement Palacos R^®^ or the experimental cement Palacos R + PLGA and the presence of the antibiotics under study (vancomycin, daptomycin, and/or linezolid). Each group had five rabbits (Table 1).

Two surgeries were performed, namely, the initial surgery and the final surgery, separated by 3 weeks (20–22 days). The surgical technique is described in an article by our study group, Azuara et al. [17]

Animals were managed in accordance with the current International Regulations on Experimental Animals (609/86/EEC and ETS 123) at the Animal Research Center of the University of Alcala. The study protocol received approval from the Committee on the Ethics of Animal Experiments of the University of Alcala (CEI UAH 2011017). The diet of the animals was available ad libitum.

### 2.4. Sample Processing: Histological and Immunohistochemical Techniques

After fixation and decalcification of the samples by Osteosoft^®^, the inclusion and cutting process was performed. Different histological techniques were performed: hematoxylin–eosin (HE) for the staining of bone and cartilage tissue, Gram staining for the staining of bacteria, and immunohistochemistry. The monoclonal antibody used to detect macrophages in rabbits was RAM-11 (DAKO ref. M633 dilution 1:50). Immunohistochemical detection of the antigen of interest was performed using the avidin–biotin complex method, using alkaline phosphatase as a tracer and Fast Red as a revealing solution. In all cases, the same biological material without primary antibody was used as a negative control. Staining was visualized under a Zeiss Axiophot optical microscope (Carl Zeiss, Oberkochen, Germany).

### 2.5. Histological Staging Methods

#### 2.5.1. Destructuring of Bone Histoarchitecture

To quantify the histological results, a new histological staging method was used, as described in the previous work by Ibarra et al. [16]. In this preliminary study, the experimental cement Palacos R + PLGA was evaluated in vivo in comparison with the commercial cement Palacos R^®^ and in the presence or absence of bacteria.

#### 2.5.2. Presence of Bacteria and the Macrophage Response

To measure the presence of bacteria and the macrophage response, we used a semiquantitative measurement method similar to that described by Remmele et al. [18]. This grading method is based on the assignment of a value estimating the percentage of the area occupied by the cells under study out of the total tissue sample. In our case, we measured the area occupied by bacteria stained with Gram stain and macrophages labeled with the monoclonal antibody RAM-11. With these percentages, we formed categories that covered the different ranges: 0 = negative, 1 = up to 25%, 2 = from 25 to 75%, and 3 = more than 75% of the total number of cells.

### 2.6. Statistical Analysis

In the evaluation of bone structure, two blinded evaluators applied the histoarchitectural staging model to five samples per rabbit, obtaining means per rabbit (*n* = 5) and per group (*n* = 6). Lin’s agreement index (Lin’s rho = 0.91, with a confidence interval of 95% (0.8–1.0)) was analyzed between the two evaluators. The semiquantitative assessments were performed in 15 microscopic fields per sample, obtaining the means per animal and per group. The normality of the averages obtained was verified by statistical tests and graphs. Given that they did not have a normal distribution, bivariate analyses were performed using the Mann–Whitney U test. All these analyses were carried out in the statistical program Prism^®^ 6.0 (GraphPad Software Incorporation, San Diego, CA, USA). The significance values are represented in the graphs as follows: * *p* < 0.05, ** *p* < 0.01, and *** *p* < 0.001.

## 3. Results

### 3.1. Evaluation of Bone Histoarchitecture: Quantitative Analysis of the Degree of Destruction

**Figure 1:**
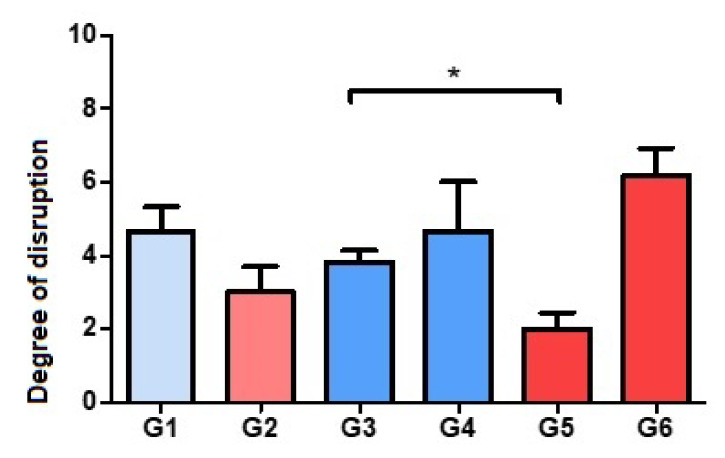
Histograms corresponding to the means and standard deviations of Groups 1 to 6. * *p* < 0.05. Degree of disruption: light (from 1 to 3), moderate (from 4 to 6), severe (from 7 to 9).

**Figure 2:**
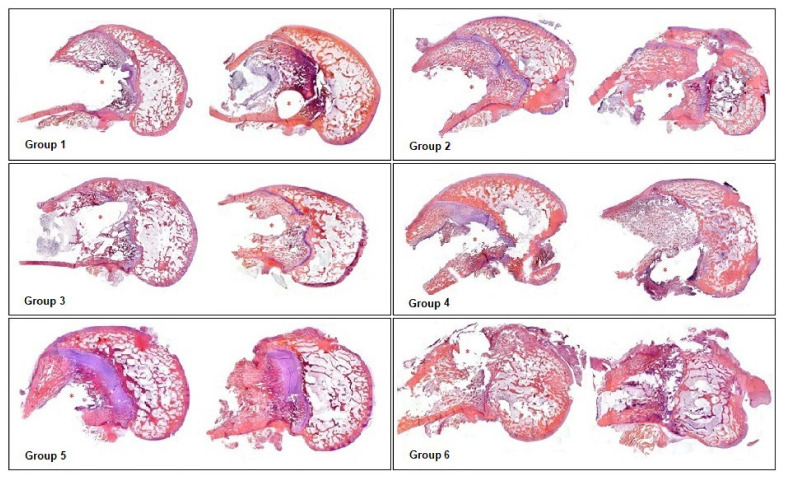
Representative panoramic images of groups of rabbits carrying contaminated rods. HE (5×). Group 1: Palacos R^®^ and daptomycin. Group 2: Palacos R + PLGA and daptomycin. Group 3: Palacos R^®^, vancomycin, and daptomycin. Group 4: Palacos R^®^, vancomycin, and linezolid. Group 5: Palacos R + PLGA, vancomycin, and daptomycin. Group 6: Palacos R + PLGA, vancomycin, and linezolid. * Location of the rod.

In the comparative analysis between groups with Palacos R^®^ cement and with Palacos R cement + PLGA, the presence of PLGA significantly improved the preservation of bone architecture when the combination of antibiotics used was vancomycin with daptomycin (Group 5). However, Palacos R + PLGA bone cement loaded with vancomycin and linezolid (Group 6) induced severe destruction of the bone architecture, so treatment with linezolid in combination with vancomycin did not preserve the bone histoarchitecture (Figure 1 and Figure 2).

### 3.2. Analysis of the Tissue Distribution of Bacteria

The distribution of *S. aureus* followed a fairly homogeneous pattern across all groups studied. The bacteria colonized the peripheral soft tissues and moved from the implant site to the physeal cartilage, subsequently occupying the epiphyseal bone and articular cartilage.

**Figure 3:**
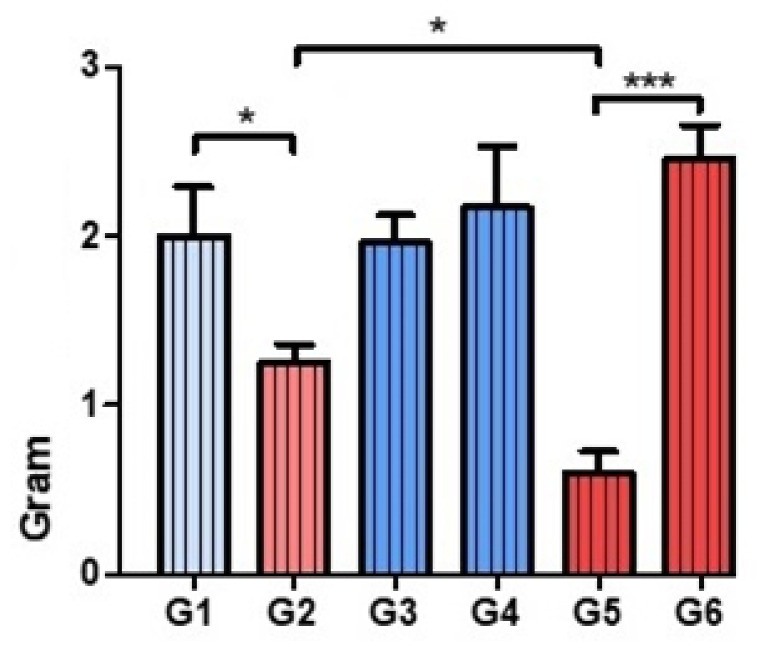
Gram staining. Histograms corresponding to the means and standard deviations of the Groups 1 to 6. * *p* < 0.05. *** *p* < 0.001.

**Figure 4:**
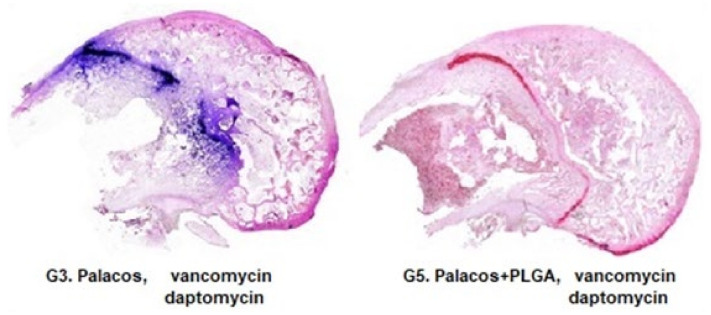
Gram-stained samples (dyed purple) of Groups 3 and 5 (5×). Note the smaller amount and smaller distribution area of bacteria in the sample of Group 5 with respect to Group 3.

**Figure 5:**
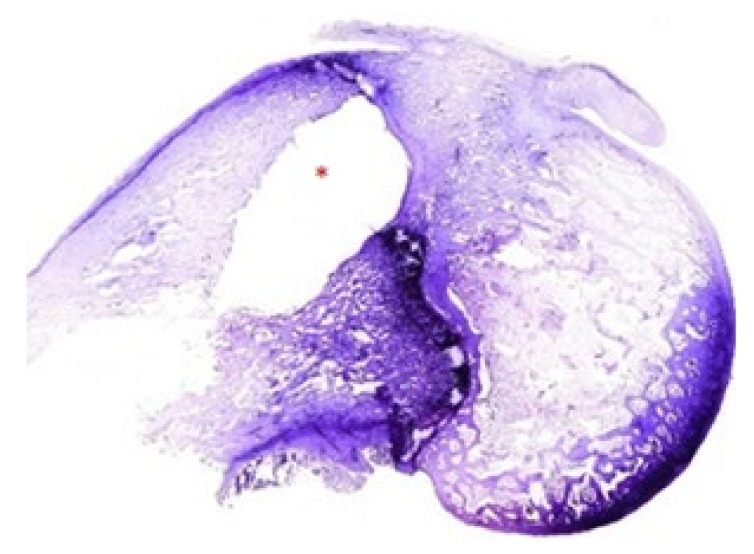
Gram staining shows the intense presence of *S. aureus* in the entire region studied in the presence of linezolid. Image corresponding to the control group (commercial cement and only linezolid). Magnification: 5×. * Imprint of contaminated rod.

The distribution of *S. aureus* was not modified by the use of Palacos R^®^ bone cement, regardless of the combination of antibiotics used (Groups 1, 3, and 4). *S. aureus* was less abundant in the presence of PLGA in all groups studied except the vancomycin + linezolid group (Group 6). This reduction was very significant when the combination of antibiotics was vancomycin + daptomycin (Group 5) (Figure 3 and Figure 4). Treatment with linezolid + vancomycin failed to control bacterial dispersion (Figure 5).

### 3.3. Assessment of the Presence of Macrophages

**Figure 6:**
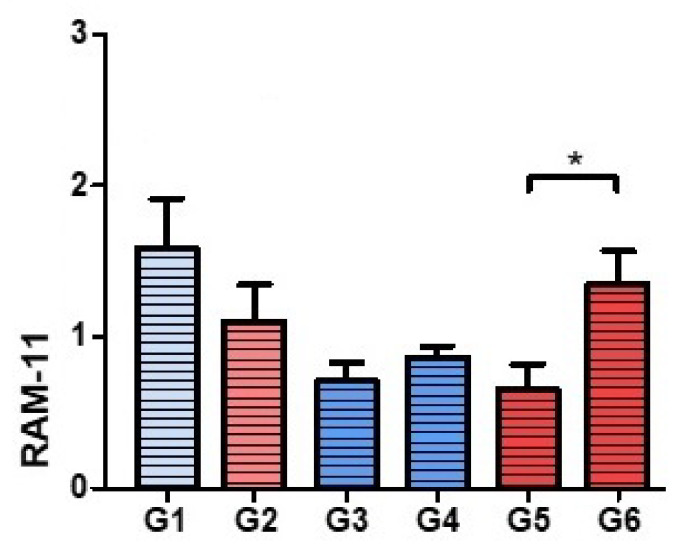
RAM 11 staining. Histograms corresponding to the means and standard deviations of Groups 1 to 6. * *p* < 0.05.

We found macrophagic cells in all groups in the capsular areas surrounding the implant, as well as in the areas with more bacteria (Figure 6). In the Palacos R + PLGA with vancomycin + linezolid group (Group 6), a greater macrophage response was associated with a greater degree of bone destruction and a high presence of bacteria (Figure 6).

## 4. Discussion

The increase in prosthetic infections caused by multiresistant bacteria has made it necessary to use new antibiotics, such as daptomycin and linezolid [7,19]. Their incorporation into bone cement has been studied in vitro [11], where they have demonstrated similar efficacy to the available gentamicin–vancomycin spacers [20]. However, there are no in vivo studies that show the behavior of the combination of these new antibiotics against bone tissue. We have published in vivo experimental studies in which we have studied the response of bone tissue to the presence of cements loaded only with linezolid [17]. In vitro studies with linezolid seem to show good antibacterial activity [11,20]. In our in vivo model [16,17], linezolid did not control bacterial growth or dispersion, showing large colonies of bacteria in the sheet extending from the implantation site to the articular cartilage under the microscope. This phenomenon was correlated with the loss of the structure of bone tissue observed in the groups that incorporated linezolid, where the tissue was so affected that, in many cases, it did not resist the action of the usual decalcifiers and routine manipulation during the processing of tissues to obtain histological samples. This observed response is in line with the toxicity that linezolid seems to exert locally and with the results of previous in vitro studies, since linezolid has been the antibiotic with the highest cytotoxicity of those studied [11]. This bone toxicity may be in line with the adverse effects observed in systemic treatment with linezolid, which include thrombocytopenia, anemia, lactic acidosis, and peripheral neuropathy [21].

However, it has been shown in vitro that daptomycin retains antibacterial properties against *S. aureus* after 5 days of incorporation into PMMA [22]. Its release is significantly higher in combination with gentamicin and tobramycin due to a synergistic effect [23]. Our working group has also previously demonstrated the synergy of daptomycin with vancomycin added to modified experimental cement (Palacos R + PLGA), where there is greater release of the combination of antibiotics than of the isolated antibiotics, as well as a biphasic release [11].

Taking into account these results, we considered it necessary to investigate the in vivo behavior of daptomycin and linezolid in combination with vancomycin, as well as the behavior of daptomycin individually loaded both in the commercial cement Palacos R^®^ and in our modified experimental cement (Palacos R + PLGA). The good bacterial control results obtained with daptomycin in our experimental model seem to be related to its ability to inhibit the formation of new biofilms [24], although it has no activity against already established biofilms [25]. In our model, the presence of *S. aureus* in the groups treated with Palacos R + PLGA cement and daptomycin was reduced to small colonies most often located in the physeal cartilage or in the peripheral soft tissues near the cartilage. Unlike the bone treated with the formulations loaded with linezolid, the bone treated with daptomycin had a well-preserved structure, and the macrophages showed a wide dispersion throughout the bone tissue, helping to reduce the presence of bacteria. This behavior may be explained by the lipid nature of daptomycin, which, as has been proven in recent studies, facilitates tissue penetration [26,27], in contrast to vancomycin, which exerts its action more locally because it has greater difficulty diffusing through bone tissue [28].

We found only one experimental study of animals treated with cement loaded with daptomycin. Rouse et al. [29] published a study in 2006 in which they treated a rat model of tibial osteomyelitis with 7.5% daptomycin, among other options, concluding that daptomycin loaded in cement is a valid option for the treatment of osteomyelitis caused by MRSA.

Our results show that Palacos R^®^ cement modified with PLGA microspheres and loaded with daptomycin allows significant preservation of the tissue structure against *S. aureus* infection. Daptomycin controls bacterial dispersion such that, in our study, at 3 weeks, there were barely any bacteria in the rabbits’ soft tissues or bone tissues, only small colonies in the physeal cartilage. Similar results were obtained with PLGA-modified Palacos R^®^ cement loaded with the synergistic combination of daptomycin and vancomycin. One of the advantages of our experimental model is that it revealed the existence of a common pattern of bacterial dispersion, which had not been described before. In the absence of bone cement loaded with antibiotics or when the antibiotic used was linezolid, at 3 weeks, the bacteria occupied the entire femoral condyle, producing extreme degradation of the bone histoarchitecture [17]. As the bacterial control by the antibiotic increased, as happened with daptomycin, the bacterial colonies were reduced to small clusters in the dispersion routes through the peripheral soft tissues to the physeal cartilage. This experimental model confirms the great affinity of *S. aureus* for peripheral soft tissues and for physeal cartilage, areas that have a concentration of many bacteria in cases of uncontrolled infection.

Our experimental model also allowed us to assess the immune response at 3 weeks after implant placement by visualizing the location of macrophages. This is because, in recent years, specific cell markers have been developed to count specific cell types in histological preparations, such as the monoclonal antibody RAM-11 for rabbit macrophages, as we have used in our model [30]. The incorporation of PLGA microspheres in commercial Palacos R^®^ cement induces changes in the behavior of macrophages. The results suggest that, although the quantitative assessments may be similar, the macrophages present a different localization depending on the type of cement used. Thus, in the groups with PLGA, these cells appeared to accumulate in the fibrous capsule without forming granulomas, remaining as macrophages, while in the groups with commercial cement Palacos R^®^, these cells, in addition to being found in the vascular lagoons, which are their natural niche, were observed in cartilage territories, a preferred place for the accumulation of bacteria. This fact is related to the lesser control of bacterial dispersion in the groups that had the commercial cement Palacos R^®^. In the presence of vancomycin, macrophages preferentially accumulated in the capsular territories around the contaminated implant and in the peripheral soft tissues, through which the bacterial migration routes are established. In contrast, in the presence of daptomycin, macrophages were dispersed throughout all tissues studied, including bone marrow and trabecular bone. This observation could be explained by the greater ease with which daptomycin penetrates tissues, given its lipophilic nature, allowing it to achieve greater bacterial control [26]. This reasoning could explain the good results obtained by the Palacos R + PLGA cement + daptomycin group, in which the two mechanisms of antibacterial action described complemented each other [11].

Due to the use of these new antibiotics, one of the concerns in the scientific community is the emergence of resistance. Although there is a clear link between the use of linezolid and the appearance of resistance despite its reduced use [31], after more than 10 years of clinical use, more than 99% of bacterial strains are still susceptible to linezolid [32]. Similarly, a recent international daptomycin surveillance program reported susceptibility rates of methicillin-sensitive *S. aureus* and MRSA of 99.9% [33].

Therefore, we can state that formulations of bone cements with the introduction of PLGA microspheres can improve the release of antibiotics, especially daptomycin, individually or in combination with vancomycin, and that these formulations loaded with daptomycin preserve the bone structure that surrounds them to a greater extent, as demonstrated in our experimental model. This may have clinical implications for the treatment of prosthetic infections caused by bacteria resistant to conventional antibiotics through the use of spacers made with these formulations of bone cement loaded with daptomycin.

## 5. Conclusions

The modification of Palacos R^®^ bone cement with PLGA microspheres and its doping with the antibiotic daptomycin in combination with vancomycin improve the tissue response to bone infection. However, the antibiotic linezolid in combination with vancomycin did not obtain good results in our model, regardless of the type of cement used.

## Figures and Tables

**Table 1:** Compositions of the experimental formulations.

Group	Cement	Antibiotic
1	Palacos R	Daptomycin
2	Palacos R + PLGA	Daptomycin
3	Palacos R	VancomycinDaptomycin
4	Palacos R	VancomycinLinezolid
5	Palacos R+PLGA	VancomycinDaptomycin
6	Palacos R+PLGA	VancomycinLinezolid

## Data Availability

The data used to support the findings of the present study are available from the corresponding author upon request.

## References

[1] Lindeque B., Hartman Z., Noshchenko A., Cruse P. (2014). Infection After Primary Total Hip Arthroplasty. Orthopedics.

[2] Chen K.-H., Tsai S.-W., Wu P.-K., Chen C.-F., Wang H.-Y., Chen W.-M. (2017). Partial component-retained two-stage reconstruction for chronic infection after uncemented total hip arthroplasty: Results of sixteen cases after five years of follow-up. Int. Orthop..

[3] Yarwood J.M., Paquette K.M., Tikh I.B., Volper E.M., Greenberg E.P. (2007). Generation of Virulence Factor Variants in Staphylococcus aureus Biofilms. J. Bacteriol..

[4] Zimmerli W., Trampuz A., Ochsner P.E. (2004). Prosthetic-joint infections. N. Engl. J. Med..

[5] Cabrita H.B., Croci A.T., de Camargo O.P., de Lima A.L.L.M. (2007). Prospective study of the treatment of infected hip arthroplasties with or without the use of an antibiotic-loaded cement spacer. Clin. Sao Paulo Braz..

[6] Anagnostakos K. (2017). Therapeutic Use of Antibiotic-loaded Bone Cement in the Treatment of Hip and Knee Joint Infections. J. Bone Jt. Infect..

[7] Tande A.J., Patel R. (2014). Prosthetic joint infection. Clin. Microbiol. Rev..

[8] Lamagni T. (2014). Epidemiology and burden of prosthetic joint infections. J. Antimicrob. Chemother..

[9] Liu C., Bayer A., Cosgrove S.E., Daum R.S., Fridkin S.K., Gorwitz R.J., Kaplan S.L., Karchmer A.W., Levine D.P., Murray B.E. (2011). Clinical Practice Guidelines by the Infectious Diseases Society of America for the Treatment of Methicillin-Resistant Staphylococcus aureus Infections in Adults and Children: Executive Summary. Clin. Infect. Dis..

[10] Pillai S.K., Wennersten C., Venkataraman L., Eliopoulos G.M., Moellering J.R.C., Karchmer A.W. (2009). Development of Reduced Vancomycin Susceptibility in Methicillin-SusceptibleStaphylococcus aureus. Clin. Infect. Dis..

[11] Parra-Ruíz F., González-Gómez A., Fernández-Gutiérrez M., Parra J., García-García J., Azuara G., De la Torre B., Buján J., Ibarra B., Duocastella-Codina L. (2017). Development of advanced biantibiotic loaded bone cement spacers for arthroplasty associated infections. Int. J. Pharm..

[12] Hashemian S.M., Farhadi T., Ganjparvar M. (2018). Linezolid: A review of its properties, function, and use in critical care. Drug Des. Dev. Ther..

[13] Gray D.A., Wenzel M. (2020). More Than a Pore: A Current Perspective on the In Vivo Mode of Action of the Lipopeptide Antibiotic Daptomycin. Antibiotics.

[14] Petty W., Spanier S., Shuster J.J., Silverthorne C. (1985). The influence of skeletal implants on incidence of infection. Experiments in a canine model. J. Bone Jt. Surg. Am..

[15] Smeltzer M.S., Thomas J.R., Hickmon S.G., Skinner R.A., Nelson C.L., Griffith D., Parr T.R., Evans R.P. (1997). Characterization of a rabbit model of staphylococcal osteomyelitis. J. Orthop. Res. Off Publ. Orthop. Res. Soc..

[16] Ibarra B., García-García J., Azuara G., Vázquez-Lasa B., Ortega M.A., Asúnsolo Á., Román J.S., Buján J., Honduvilla N.G., De La Torre B. (2019). Polylactic-co-glycolic acid microspheres added to fixative cements and its role on bone infected architecture. J. Biomed. Mater. Res. Part B Appl. Biomater..

[17] Azuara G., García-García J., Ibarra B., Parra-Ruiz F.J., Asúnsolo A., Ortega M.A., Vázquez-Lasa B., Buján J., San Román J., de la Torre B. (2019). Experimental study of the application of a new bone cement loaded with broad spectrum antibiotics for the treatment of bone infection. Rev. Espanola Cirugia Ortop. Traumatol..

[18] Remmele W., Schicketanz K.-H. (1993). Immunohistochemical determination of estrogen and progesterone receptor content in human breast cancer: Computer-assisted image analysis (QIC score) vs. subjective grading (IRS). Pathol. Res. Pract..

[19] Ariza J., Cobo J., Baraia-Etxaburu J., de Benito N., Bori G., Cabo J., Corona P., Esteban J., Horcajada J.P., Lora-Tamayo J. (2017). Executive summary of management of prosthetic joint infections. Clinical practice guidelines by the Spanish Society of Infectious Diseases and Clinical Microbiology (SEIMC). Enferm. Infecc. Microbiol. Clin..

[20] Anagnostakos K., Kelm J., Grün S., Schmitt E., Jung W., Swoboda S. (2008). Antimicrobial properties and elution kinetics of linezolid-loaded hip spacers in vitro. J. Biomed. Mater Res. B Appl. Biomater..

[21] Beibei L., Yun C., Mengli C., Nan B., Xuhong Y., Rui W. (2010). Linezolid versus vancomycin for the treatment of Gram-positive bacterial infections: Meta-analysis of randomised controlled trials. Int. J. Antimicrob. Agents.

[22] Kuechle D.K., Landon G.C., Musher D.M., Noble P.C. (1991). Elution of Vancomycin, Daptomycin, and Amikacin From Acrylic Bone Cement. Clin. Orthop. Relat. Res..

[23] Kaplan L., Kurdziel M., Baker K., Verner J. (2012). Characterization of Daptomycin-loaded Antibiotic Cement. Orthopedics.

[24] Eick S., Hofpeter K., Sculean A., Ender C., Klimas S., Vogt S., Nietzsche S. (2017). Activity of Fosfomycin- and Daptomycin-Containing Bone Cement on Selected Bacterial Species Being Associated with Orthopedic Infections. BioMed Res. Int..

[25] Stefani S., Campanile F., Santagati M., Mezzatesta M.L., Cafiso V., Pacini G. (2015). Insights and clinical perspectives of daptomycin resistance in Staphylococcus aureus: A review of the available evidence. Int. J. Antimicrob. Agents.

[26] Grillon A., Argemi X., Gaudias J., Ronde-Ousteau C., Boeri C., Jenny J.-Y., Hansmann Y., Lefebvre N., Jehl F. (2019). Bone penetration of daptomycin in diabetic patients with bacterial foot infections. Int. J. Infect. Dis..

[27] Mescola A., Ragazzini G., Alessandrini A. (2020). Daptomycin Strongly Affects the Phase Behavior of Model Lipid Bilayers. J. Phys. Chem. B.

[28] Bue M., Tøttrup M., Hanberg P., Langhoff O., Birke-Sørensen H., Thillemann T.M., Søballe K. (2018). Bone and subcutaneous adipose tissue pharmacokinetics of vancomycin in total knee replacement patients. Acta Orthop..

[29] Rouse M.S., Piper K.E., Jacobson M., Jacofsky D.J., Steckelberg J.M., Patel R. (2006). Daptomycin treatment of Staphylococcus aureus experimental chronic osteomyelitis. J. Antimicrob. Chemother..

[30] Kanazawa T., Soejima T., Murakami H., Inoue T., Katouda M., Nagata K., Wang J.-W., Fong C.-Y., Su Y.-S., Yu H.-N. (2006). An immunohistological study of the integration at the bone-tendon interface after reconstruction of the anterior cruciate ligament in rabbits. J. Bone Jt. Surg. Br. Vol..

[31] Baos E., Candel F.J., Merino P., Pena I., Picazo J.J. (2013). Characterization and monitoring of linezolid-resistant clinical isolates of Staphylococcus epidermidis in an intensive care unit 4 years after an outbreak of infection by cfr-mediated linezolid-resistant Staphylococcus aureus. Diagn. Microbiol. Infect. Dis..

[32] Flamm R.K., Mendes R., Ross J.E., Sader H., Jones R.N. (2013). An international activity and spectrum analysis of linezolid: ZAAPS Program results for Diagn. Microbiol. Infect. Dis..

[33] Sader H.S., Flamm R.K., Jones R.N. (2013). Antimicrobial activity of daptomycin tested against Gram-positive pathogens collected in Europe, Latin America, and selected countries in the Asia-Pacific Region (2011). Diagn. Microbiol. Infect. Dis..

